# Extended spectrum beta lactamase producing *Escherichia coli* eustachian valve infective endocarditis

**DOI:** 10.1016/j.amsu.2021.102705

**Published:** 2021-08-10

**Authors:** Branden Ireifej, David Song, Pradeeksha Mukuntharaj, Tasur Seen, Talal Almas, XiongBin Lin, M. Ali Kanawati, Yasar Sattar

**Affiliations:** a Icahn School of Medicine at Mount Sinai - Elmhurst Hospital Center, Elmhurst, NY, USA; b Grant Medical College and Sir JJ Group of Hospitals, Mumbai, India; c RCSI University of Medicine and Health Sciences, Dublin, Ireland; d Division of Cardiology, West Virginia University, Morgantown, WV, USA

**Keywords:** Infective endocarditis, *Escherichia coli* (*E. coli)* ESBL, Eustachian valve, End-stage renal disease (ESRD), Transesophageal echocardiogram (TEE)

## Abstract

Endocarditis is an infection of the endocardium caused by a multitude of bacteria, including *S. aureus*, viridans streptococci, S. bovis, or S. epidermidis, among others. It can cause a variety of physical findings, including new onset murmur, Osler nodes, and Janeway lesions. Endocarditis is diagnosed with multiple positive blood cultures with transesophageal echocardiogram (TEE) showing valvular vegetations. In this article, we present a 47 year old female with a history of ESRD on dialysis who presented with a bleeding fistula found to be in septic shock. Diagnosis of eustachian valve endocarditis with *E. Coli* ESBL was made through positive blood cultures as well as using TEE. She was started on IV meropenem for seven days, to which the patient completed and eventually was discharged home with resolution of symptoms.

## Highlights

● There are only a few case reports in literature on *E. Coli* ESBL induced Eustachian valve endocarditis, which makes it an extremely rare presentation or a frequently unnoticed condition
● The diagnosis of endocarditis is made through Duke's criteria where you have positive blood cultures and echocardiogram revealing vegetation
● Persistent *E. coli* bacteremia in elderly in the absence of cardiac risk factors while on appropriate antibiotic therapy should consider *E. Coli* ESBL induced Eustachian valve endocarditis as a differential

## 1 Introduction

Infective endocarditis (IE) refers to infection of the endocardial surface of the heart; usually involving one or more heart valves or infection of an intracardiac device. IE may be acquired in the community or in the setting of a hospital environment. Community-associated IE develops in the absence of recent contact with a hospital setting, with diagnosis established within 48 hours of hospital admission. Health care-associated IE develops in the context of recent contact with a hospital setting, with onset of symptoms ≥48 hours after hospitalization. Bactericidal agents are necessary for effective treatment of endocarditis. Presentation of endocarditis is broad, and can include fever, new murmur, Roth spots, Janeway lesions, glomerulonephritis, and splinter hemorrhages on the nail bed. It can arise from bacteria, fungi, or secondary to other causes (malignancy, hypercoagulable states, systemic lupus).

In this article, we present a rare case of Eustachian Valve Endocarditis caused by ESBL producing E. Coli in a patient with ESRD on dialysis, initially presenting with septic shock. In addition, this work has been reported in accordance with SCARE [1].

## 2 Case

A forty seven year old female with a past medical history of hypertension (HTN), Type II Diabetes Mellitus (TIIDM), Hyperlipidemia (HLD), seizures, and ESRD on hemodialysis (HD) presented to the emergency department from dialysis with a bleeding arterio-venous graft (AVG) on the right upper arm. The patient presented with an altered mental status alert oriented to only person and not to place or time, fever of 103.0 F, hypotensive to 74/45, sinus tachycardia to 120s, and tachypnea to 23. On arrival, the patient was in septic shock, requiring norepinephrine and given vancomycin and cefepime for broad spectrum antibiotic coverage. Patient had a femoral arterial line placed along with two peripheral intravenous lines (IV). Patient was admitted to the Intensive Care Unit (ICU) for further monitoring and coverage.

In the ICU, the patient received HD, vancomycin, cefepime, levetiracetam (for seizure control), insulin glargine at night along with meal time correctional insulin lispro, and at most required 4 L of oxygen via nasal cannula. Vascular surgery, neurology, nephrology, and infectious disease were all consulted. Vascular surgery was consulted for suspected graft infection from AVG, but had low suspicion for graft infection. Neurology recommended levetiracetam for seizure control throughout the hospital course. Nephrology was consulted for continuation of dialysis for the patients ESRD. Infectious disease was consulted for work up of suspected bacteremia.

Left upper extremity (LUE) ultrasound of AVG showed no signs of abscess, and white blood cell (WBC) count trended up throughout the course of hospital. Platelet count trended down in the setting of septic shock requiring one pool of platelets during the hospital course.

**Fig. 1:**
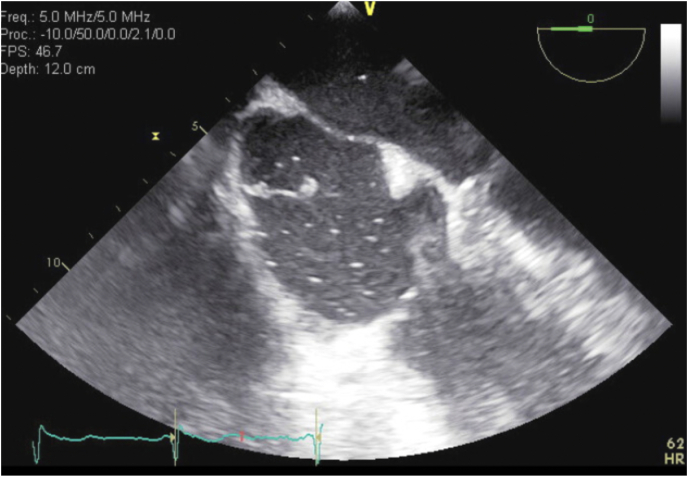
Transesophageal echocardiogram (TEE) confirming vegetation on the Eustachian valve, likely from *E. coli* ESBL.

Blood cultures were positive for E. coli ESBL in two bottles, three days after collection. The patient's antibiotic coverage was switched to IV meropenem 500 mg every day for seven days and vancomycin and cefepime were discontinued after the blood cultures revealed that the ESBL E. Coli was sensitive to meropenem. Daily blood cultures were ordered and followed until resolution of bacteremia. Transthoracic Echocardiography (TTE) was performed, which revealed an echodensity in the right atrium. TEE further confirmed vegetation on the eustachian valve, likely from *E. coli* ESBL **(**Fig. 1**)**. No surgical intervention was recommended at this time, rather only to continue with meropenem.

Patient was downgraded to the step down unit once she was hemodynamically stable. As per infectious disease, a computed tomography abdomen and pelvis and computed tomography chest with contrast was completed to determine if any occult source of infection existed. All imaging came back unremarkable for any source of occult infection. In addition, TTE was repeated which revealed decrease in the echodensity of the right atrium suggesting improvement on antibiotics. Patient was subsequently transferred to non-teaching service for a full 6-week course of IV antibiotics for ESBL *E. coli* endocarditis. Patient was subsequently transferred to non-teaching service for a full 6-week course of IV antibiotics for ESBL *E. coli* endocarditis.

Based on the findings listed above, it is presumed this patient had infective endocarditis based on the Modified Duke's criteria (Table 1) and overall clinical picture.

**Table 1:** Modified Duke's infective endocarditis criteria [14].

Criteria	Definitive Infective Endocarditis	Possible Infective Endocarditis	Not Infective Endocarditis
Pathologic			
Histologic	Vegetation or intracardiac abscess present, confirmed by histology showing active endocarditis	Short of definite, but not rejected	No pathologic evidence of infective endocarditis with antibiotic therapy for 4 days or less
**OR**			
Bacteria	Demonstrated by culture or histology in a vegetation, or in a vegetation that has embolized, or in an intracardiac abscess	Short of definite, but not rejected	No pathologic evidence of infective endocarditis with antibiotic therapy for 4 days or less
**Clinical - Any one of the following**			
Major criteria	2	Does not apply	Resolution of manifestations of endocarditis, with antibiotic therapy for 4 days or less, or firm alternate diagnosis for manifestations of endocarditis. Does not meet criteria for possible infective endocarditis
Minor criteria	5	3	
Major and Minor criteria	1 major +3 minor	1 major and 1 minor	
Major criteria			
**A. Supportive laboratory evidence** Typical microorganism for infective endocarditis from two separate blood cultures: viridans streptococci, *Staphylococcus aureus*, Streptococcus bovis, HACEK group (Haemophilus spp. Actinobacillus actinomycetemcomitans, Cardiobacterium hominis, Eikenella spp., and Kingella kingae) or Community-acquired enterococci, in the absence of a primary focus			
Persistently positive blood culture, defined as recovery of a microorganism consistent with infective endocarditis from blood cultures drawn more than 12 hours apart or Persistently positive blood culture, defined as recovery of a microorganism consistent with infective endocarditis from all of three or a majority of four or more separate blood cultures, with first and last drawn at least 1 hour apart.			
Single positive blood culture for Coxiella burnetti or phase I antibody titer >1:800			
**B. Evidence of endocardial involvement** Echocardiogram supportive of infective endocarditis. **1. Type of study** **TEE is recommended as the first test in the following patients**: a) prosthetic valve endocarditis; or b) those with at least “possible” endocarditis by clinical criteria; or c) those with suspected complicated endocarditis, such as paravalvular abscess. TTE recommended as first test in all other patients **2**. **Definition of positive findings**: oscillating intracardiac mass, on valve or supporting structures, or in the path of regurgitant jets, or on implanted material, in the absence of an alternative anatomic explanation or myocardial abscess or new partial dehiscence of prosthetic valve			
**C. New valvular regurgitation (increase or change in pre-existing murmur not sufficient).**			
Minor Criteria			
Predisposing heart condition or Intravenous drug use			
Fever≥38C (100.4 F)			
Vascular phenomena: major arterial emboli, septic pulmonary infarcts, mycotic aneurysm, intracranial hemorrhage, conjunctival hemorrhage, Janeway lesions			
Immunologic phenomena: glomerulonephritis, Osler's nodes, Roth spots, rheumatoid factor			
Positive blood culture not meeting major criterion as noted previously (Excluding single positive cultures for coagulase-negative staphylococci and organisms that do not cause endocarditis) or serologic evidence of active infection with organism consistent with infective endocarditis			

## 3 Discussion

Right sided valvular involvement in IE has been well-described, but lesions affecting the eustachian valve are distinctly rare [2]. The eustachian valve (EV; also known as the valve of inferior vena cava) is an embryological remnant of the sinus venosus, directing oxygenated fetal blood from the inferior vena cava (IVC) across the foramen ovale, and into the left atrium. In adults, it is non-functional and is considered a benign structure. It is easily visualized on routine ultrasound and is seen in approximately 25% of individuals as an elongated membranous structure extending from the IVC to the border of the fossa ovalis, which can become a focus of infection in rare cases [3].

Only 37 cases of EV endocarditis (EVE) have been reported from 1986 to 2018, 46% of which was caused by IV drug use and 24% by indwelling IV [4,5]. The usual predisposing factor for EVE is intravenous drug use (IVDU). Other causes include indwelling catheters, rheumatic heart disease, pacemaker wires, and immunologic compromise (such as that caused by chronic alcoholism or the human immunodeficiency virus, diabetes) [6]. The most common causative organism in IVDU is *Staphylococcus aureus*. Other organisms, not specifically associated with IVDU, include *Staphylococcus hominis*, *Enterobacter cloacae*, *Escherichia coli*, *Proteus vulgaris*, *Streptococcus viridans, Klebsiella pneumonia*, and *Actinomyces israelii* [7]. The patient in our case had a past medical history of HTN, TIIDM, HLD, seizures, and ESRD on dialysis which signifies the importance of recognizing risk factors other than IVDU in the pathogenesis of EVE.

*E.coli* native valve endocarditis (NVE) is extremely rare despite being a common cause of bacteremia. In our case, the patient had no prior degenerative valvular condition but had TIIDM and ESRD on HD and presented with septic shock that could be attributed to *E. coli* bacteremia (urosepsis or pyelonephritis). A patient with multiple risk factors, presence of septic shock, persistent fever, and blood cultures positive for E. coli ESBL, there was a high index of suspicion for IE.

TTE plays a key role for the diagnosis of IE and must be performed as soon as IE is suspected. TEE is performed when TTE is positive or non-diagnostic [8,10]. In a retrospective analysis by Biswas A et al., 27 high risk patients for IE that underwent TTE and TEE examinations were studied and only 29.6% of these patients were found to have vegetation on TTE. This study recommended TEE as the initial diagnostic tool for high risk patients without need for TTE [9]. In our case, the patient had high risk features and therefore warranted immediate TEE to rule out IE which confirmed vegetation on the eustachian valve, likely from *E. coli* ESBL, given the positive blood culture tests.

Carbapenems are the drug of choice for severe infections due to ESBL-producing *Enterobacteriaceae*. The presence of ESBL makes *E. coli* resistant to third and fourth-generation cephalosporins, monobactams, fluoroquinolones, tetracyclines, and aminoglycosides [11,12]. Surgical treatment is associated with high mortality and reserved for heart failure due to valvular regurgitation and uncontrolled infection due to periannular extension or difficult-to-treat microorganisms [13]. Analysis of the ICE-Prospective Cohort Study (ICE-PCS) database suggested no major survival benefit with additional cardiac surgery or with combination antibiotic therapy. In our case, the patient was switched to IV meropenem 500 mg daily for 1 week and surgical intervention was deferred as the patient began improving clinically. After repeat CT chest with abdomen and pelvis were unremarkable for occult source of infection, the patient was shifted to the non-teaching service for a full 6-week course of IV antibiotics for ESBL *E. coli* endocarditis as recommended by infectious disease.

The patient discussed in this case report satisfied two major modified Duke criteria including positive blood cultures and evidence of endocardial involvement with TEE significant for a vegetation on the eustachian valve thus proving the diagnosis of infective endocarditis (Table 1).

In our case, the patient likely developed pyelonephritis or urosepsis secondary to ESRD on HD which was the primary event triggering *E. Coli* sepsis as there were no predisposing heart conditions or IVDU. Worsening hemodynamic status initially was due to *E. Coli* ESBL not responding to vancomycin and cefepime. Improvement in hemodynamic status and repeat imaging unremarkable for any source of infection including CT chest with abdomen and pelvis, and TTE while on meropenem, further confirmed *E. Coli* sepsis.

The differential diagnosis for persistent fever and septic shock in a patient on broad spectrum antibiotics with high risk factors should include IE. Persistent *E. coli* bacteremia in the elderly in the absence of cardiac risk factors should indicate the need for prompt echocardiography to rule out IE, as in this case report. This case report illustrates the need to consider *E. Coli* ESBL induced eustachian valve endocarditis in patients with septicemia (urosepsis), having persistent fever while on appropriate antibiotic therapy.

## 4 Conclusion

Herein, we have reported a successfully treated case of distinctly rare eustachian valve IE due to ESBL *E. coli* in a patient with a history of ESRD on HD. Because of the rising prevalence of elderly population with degenerative valvular disease and increased frequency of immunosuppressive treatment, clinicians should consider early ESBL *E. coli* coverage in patients without clinical improvement on antibiotics or patients with immunocompromised state.

## Provenance and peer review

Not commissioned, externally peer reviewed.

## Declaration of competing interest

None.
